# Estimated impact of long‐acting injectable PrEP in South Africa: a model comparison analysis

**DOI:** 10.1002/jia2.26453

**Published:** 2025-07-02

**Authors:** Sarah E. Stansfield, Mia Moore, Lise Jamieson, Gesine Meyer‐Rath, Leigh F. Johnson, David Kaftan, Anna Bershteyn, Jennifer Smith, Valentina Cambiano, Loveleen Bansi‐Matharu, Andrew Phillips, Jesse Heitner, Ruanne V. Barnabas, Brett Hanscom, Deborah J. Donnell, Marie‐Claude Boily, Dobromir Dimitrov

**Affiliations:** ^1^ Fred Hutchinson Cancer Center Seattle Washington USA; ^2^ Health Economics and Epidemiology Research Office Faculty of Health Sciences University of the Witwatersrand Johannesburg South Africa; ^3^ The South African Department of Science and Innovation/National Research Foundation Centre of Excellence in Epidemiological Modelling and Analysis (SACEMA) Stellenbosch University Stellenbosch Republic of South Africa; ^4^ Department of Global Health Boston University School of Public Health Boston Massachusetts USA; ^5^ University of Cape Town Rondebosch South Africa; ^6^ New York University Grossman School of Medicine New York New York USA; ^7^ University College London London UK; ^8^ Division of Infectious Diseases Massachusetts General Hospital, Harvard Medical School Boston Massachusetts USA; ^9^ MRC Centre for Global Infectious Disease Analysis School of Public Health, Imperial College London London UK

**Keywords:** adherence, CAB‐LA, HIV prevention, modelling, pre‐exposure prophylaxis, South Africa

## Abstract

**Introduction:**

Long‐acting injectable cabotegravir (CAB‐LA) demonstrated superiority to daily tenofovir disoproxil fumarate/emtricitabine (TDF/FTC) for human immunodeficiency virus (HIV) pre‐exposure prophylaxis (PrEP) in two clinical trials. This analysis projects the impact of expanding PrEP coverage with CAB‐LA in South Africa between 2022 and 2042.

**Methods:**

Three independently calibrated models of HIV transmission in South Africa (Synthesis, EMOD‐HIV, Thembisa) projected HIV acquisitions and effective coverage (average PrEP coverage across exposure groups, weighted by HIV incidence in the absence of PrEP in each group) over 20 years under multiple scenarios of PrEP expansion compared to no PrEP expansion. PrEP expansion scenarios differed in targeted overall coverage, speed of expansion, coverage of high‐exposure groups, and relative coverage of women and men.

**Results:**

Achieving 5% PrEP coverage with CAB‐LA by 2032 prioritizing high‐exposure groups resulted in 49% (Synthesis), 18% (EMOD‐HIV), and 8% (Thembisa) effective coverage and averted a median of 43%, 29% and 10% of new HIV acquisitions, respectively. Similar expansion with TDF/FTC resulted in lower impact by 19 percentage points (pp), 18pp and 3pp, respectively. Increasing CAB‐LA coverage to 15% led to an additional 7pp, 12pp and 16pp, respectively, of HIV acquisitions averted. Achieving 5% CAB‐LA coverage expanding to women only resulted in a lower impact by 16pp (Synthesis) and 13pp (EMOD‐HIV), and a higher impact by 2pp (Thembisa). Scenarios with similar effective coverage resulted in comparable impact estimates across models.

**Conclusions:**

Offering CAB‐LA in South Africa may substantially impact the HIV epidemic based on these projections. Effective coverage proved to be a good predictor of intervention effectiveness.

## 1 INTRODUCTION

Two HIV Prevention Trials Network (HPTN) clinical studies demonstrated that pre‐exposure prophylaxis (PrEP) with long‐acting injectable cabotegravir (CAB‐LA) was effective at preventing HIV among transgender women and cisgender men who have sex with men (MSM) (HPTN 083) [1] and among cisgender women (HPTN 084) [2]. CAB‐LA requires an injection every 8 weeks and is generally preferred to daily oral tenofovir disoproxil fumarate/emtricitabine (TDF/FTC) [3, 4, 5]. Ninety‐six percent of US HPTN 083 and 78% of HPTN 084 participants chose CAB‐LA over TDF/FTC in the open‐label extension to the trials [6, 7]. The World Health Organization (WHO) recommends CAB‐LA use as part of combination prevention approaches [8]. Other long‐acting PrEP products are in the final stage of testing, with twice‐yearly injections of Lenacapavir recently demonstrating substantially better efficacy than oral PrEP regimens in preventing HIV acquisition among women in South Africa and Uganda [9].

The impact of new HIV prevention products depends on clinical efficacy and uptake, adherence, and persistence with the product by high‐incidence groups. Completed oral PrEP efficacy studies consistently showed low effectiveness among heterosexual women in Africa [10]. A modelling study estimated 96% effectiveness in women if daily oral TDF/FTC PrEP was used as prescribed but 60% effectiveness if two pills were taken per week, suggesting that there is less forgiveness for missed doses for women than MSM [11]. There are many barriers that put women at a higher likelihood for acquiring HIV and make it difficult to incorporate frequent TDF/FTC pill taking into their lives, including significant gender inequality, high levels of intimate partner violence, and gendered responsibilities for child and household care [12, 13]. In 2016, 5 years after the introduction of oral PrEP, uptake and knowledge of PrEP in South African healthcare clinics remained low [14]. Stigma and misinformation around PrEP persist, especially for female sex workers (FSWs) [15] and adolescent girls and young women (AGYW) [16]. Persistence on oral PrEP is low in AGYW, with only 31% of participants in a demonstration project in Kenya and South Africa refilling their PrEP prescriptions after 1 month [17]. As a result, the HIV incidence in South Africa remains high, with incidence levels of 4.5/100 person years (PYs) reported in young women seeking contraception between 2015 and 2018 [18] and 4.6/100 PYs in FSWs in 2019 [19]. It is expected that the CAB‐LA PrEP regimen will be easier to follow and adhere to [2], as well as more discreet with a longer duration of protection. These are all factors women have expressed desire for [20] and may help overcome some barriers in PrEP use. Everyone is eligible for PrEP with current guidelines in South Africa.

Several modelling studies have examined the impact of CAB‐LA in sub‐Saharan Africa. One model estimated that if CAB‐LA use increased to 46% of those with a PrEP indication (2.6% of the adult population), HIV incidence would decline by 29% over the next 20 years [21]. The same analysis predicted that while CAB‐LA rollout may increase integrase‐inhibitor drug resistance, overall AIDS‐related deaths would decrease [21]. A model of providing CAB‐LA to key populations, including FSWs, MSM, AGYW, and adolescent boys and young men, projected it would avert more HIV acquisitions compared to providing TDF/FTC only [22]. Another analysis using the same model predicted that offering CAB‐LA to pregnant and breastfeeding women (54% coverage) in sub‐Saharan Africa could reduce their HIV acquisitions by 41% and by 30% in their breastfed children [23].

The HPTN Modelling Centre (https://hptnmodelling.org/) and the HIV Modelling Consortium (http://hivmodeling.org/) collaborated to project population‐level impacts of CAB‐LA PrEP scale up by inviting teams to participate in model comparison projects, using specific model parameters and scenarios. Here, we present results from this collaborative project examining PrEP scale up in cisgender populations in South Africa (due to current data limitations we did not include transgender populations). We compare projections from three independently parameterized and calibrated mathematical models of HIV transmission in South Africa employing different assumptions about the distribution of HIV acquisition risk and exploring different PrEP prioritization strategies. We find commonalities and identify key drivers of discrepancies in modelling results to help results’ interpretation and reusability. Our goal is to support readers in their decision about which modelling setup most closely relates to their epidemic settings and which intervention scenarios are feasible to implement. Based on this analysis, we propose a novel evaluation metric which should be useful in future decision‐making.

## 2 METHODS

Three independently developed and calibrated models of the HIV epidemic in South Africa were included in this comparison: Synthesis [21, 24], EMOD‐HIV [25, 26, 27] and Thembisa [22]. These models’ projections (without PrEP) have been previously compared and found to be fairly consistent at the start of our simulated time frame with more variability at the end [28]. Each model used a detailed, mechanistic simulation of a synthetic population. Synthesis and EMOD‐HIV simulated the sexual activity and HIV status of individuals, whereas Thembisa tracked HIV incidence within fine‐grained sub‐populations. TDF/FTC effectiveness, PrEP indication criteria, antiretroviral therapy (ART) use, and demographic and sexual behaviour parameters were specific to each model (Table 1 and Supporting Information). CAB‐LA effectiveness and persistence were based on results from HPTN 083 and HPTN 084 [1, 2, 29].

**Table 1:** Model characteristics

Model characteristics	Synthesis model	EMOD‐HIV model	Thembisa model
Model structure	Stochastic individual‐based	Stochastic individual‐based	Deterministic compartmental
Modelled population	Heterosexuals, FSW	Heterosexuals, FSW	Heterosexuals, FSW, men who have sex with men (MSM)
Baseline TDF/FTC coverage	0.76%	2.20%	0.05%
TDF/FTC effectiveness	Mean 70%^a^	58%	85% MSM 65% remaining population
TDF/FTC discontinuation rate^b^	0.01−0.05 per 3 months	0.31 per 3 months	0.02 per 3 months
CAB‐LA effectiveness	95% (80% runs) 90% (20% runs)	95%	95% women 91% men
CAB‐LA discontinuation rate^b^	0.01−0.05 per 3 months	0.02 per 3 months	
Proportion in each HIV exposure group^c^	High exposure: 8%, 16%, 24% of adults aged 15–65^d^ with 5%, 10% and 15% PrEP coverage, respectively	High exposure: 3% Medium exposure: 17% Low exposure: 80%	High‐exposure group: women: 22% men: 32%
Likelihood of HIV acquisition ratio between exposure groups^e^, ^f^	1:34, 1:18, 1:17 Low: High‐exposure group^d^ with 5%, 10% and 15% PrEP coverage, respectively	1:6:9 Low: Medium: High‐exposure group	1:5 Low: High‐exposure group
PrEP allocation by HIV exposure group (Prioritization model scenarios)	PrEP only used by those in the high‐exposure group in any given 3‐month period^d^	High‐exposure group covered before any of the medium‐exposure group is covered	High‐exposure group 3x more likely to take up PrEP than low‐exposure group

*Note*: For full table, see Table S2.1.

Abbreviations: CAB‐LA, long‐acting injectable cabotegravir; FSW, female sex worker; HIV, human immunodeficiency virus; MSM, men who have sex with men; PrEP, pre‐exposure prophylaxis; TDF/FTC, tenofovir disoproxil fumarate/emtricitabine.

^a^ Effectiveness was defined as the combination of product efficacy and adherence. There was a 20% chance efficacy = 90% and an 80% chance efficacy = 95%. Adherence was > 80% in 90% of individuals, resulting in mean effectiveness of approximately 70%.

^b^ Discontinuation rates despite continued risk of HIV acquisition.

^c^ HIV exposure groups are defined by differences in sexual activity and mixing patterns which affect the likelihood to acquire HIV.

^d^ The model uses specific PrEP indication criteria which we use to define the high‐exposure group. This estimate represents the mean over multiple stochastic runs (see Supporting Information).

^e^ Relative difference in annual HIV incidence between groups in the absence of PrEP.

^f^ These are model outputs not inputs.

### 2.1 PrEP scenarios and prioritization

The baseline scenario maintained TDF/FTC use and ART coverage at or near baseline levels. PrEP expansion scenarios varied the magnitude and pace of expansion and PrEP prioritization (Table 2). PrEP expansion was initiated in 2022 and increased the overall PrEP coverage in the total adult population in South Africa without HIV. Either CAB‐LA or TDF/FTC daily pills were offered to all users. PrEP was either prioritized to high HIV exposure groups or distributed proportionally across the entire population without HIV. Each model prioritized high‐exposure groups differently. In EMOD‐HIV, PrEP was expanded first among those in the high‐exposure group (as defined in the Supporting Information), then to the medium‐exposure group. In Thembisa, PrEP was available to all, but the high‐exposure group was three times more likely to initiate PrEP than the remaining population. In Synthesis, PrEP expansion was prioritized according to one of 10 different criteria sets, which were then sorted by the overall population PrEP coverage achieved in each simulation. Because HIV prevalence is higher in women compared to men in South Africa [30], we performed separate analyses of offering PrEP to women only versus to the full population, applying the same population coverage targets. See Supporting Information for full details.

**Table 2:** PrEP expansion intervention details

Intervention components	Scenarios simulated
PrEP coverage of the population aged 15 years or older, without HIV	5%, 10%, 15% and 20% (5−15% Synthesis) *Simulations were constrained to not exceed the targeted coverage by more than 1 percentage point*.
Time to achieve targeted PrEP coverage	5 and 10 years (by 2027 and by 2032)
PrEP distribution	Prioritized PrEP expansion: PrEP is used preferentially by those in higher HIV exposure groups Proportional PrEP expansion: All individuals without HIV have equal chances of using PrEP
	Women and men Women only

Abbreviation: PrEP, pre‐exposure prophylaxis.

### 2.2 Metrics of impact

The population‐level impact was assessed using the *cumulative fraction of new HIV acquisitions averted over 20 years* (calculated as the complement of the cumulative number of new HIV acquisitions occurring between 2022 and 2042 in each PrEP expansion scenario divided by the cumulative number of HIV acquisitions in the baseline scenario). Population‐level efficiency was measured as the *number needed to treat (NNT)* to prevent one HIV acquisition and was calculated as the number of additional PYs on PrEP in each PrEP expansion scenario (compared to baseline) divided by the number of acquisitions averted (difference from baseline) between 2022 and 2042. We also calculated the *effective coverage*, defined as average PrEP coverage across all exposure groups, weighted by the HIV incidence in the absence of PrEP in each exposure group (Equation S1). The size of each HIV exposure group was calculated at baseline in 2022. “Estimate” and “project” here refer to model outputs not in the statistical sense. We report means (with 95% confidence intervals of the means) of simulation replicates (for stochastic models) or parameter sets (for the deterministic model). Simulations with prioritized PrEP expansion and PrEP coverage levels achieved by 2032 are presented in the main text and other results are shown in the Supporting Information unless explicitly noted.

### 2.3 Role of the funding source

The study funding source had no role in the design or interpretation of the study or in the writing of the manuscript.

## 3 RESULTS

### 3.1 Model projections for the baseline scenarios assuming limited TDF/FTC use

**Figure 1:**
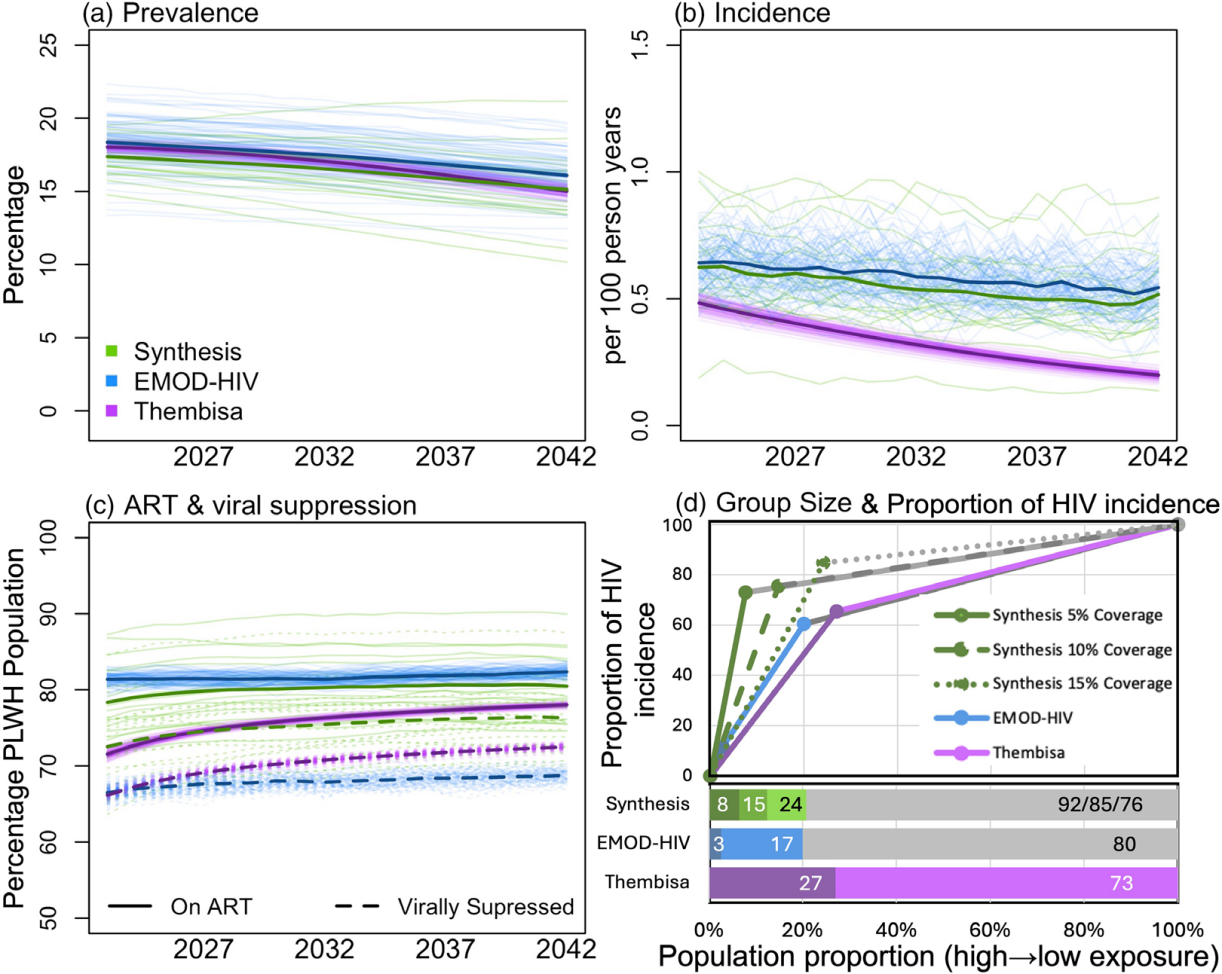
**Baseline model projections by Synthesis (green), EMOD‐HIV (blue) and Thembisa (purple)**. (a) HIV prevalence. (b) HIV incidence. (c) ART coverage and viral suppression among the people living with HIV (PLWH) population. (d) Percentage of people by HIV exposure group and corresponding proportion of HIV incidence in each group calculated at baseline in 2022. Dark colours show high‐exposure groups, lighter colours show medium‐exposure groups (not present in Synthesis) and grey shows low‐exposure groups (not present in Thembisa). Synthesis with 5% pre‐exposure prophylaxis (PrEP) coverage is shown as a solid line, Synthesis with 10% PrEP coverage is shown with a dashed line and Synthesis with 15% PrEP coverage is shown with a dotted line. Bold lines in (a−c) show mean values of multiple simulation replicates (for stochastic models) or parameter sets (for the deterministic model), lighter lines show scenarios directly. Viral suppression definitions are provided in Table S2.1. Abbreviation: ART, antiretroviral therapy.

In the baseline scenario, each model projected a slight decline in HIV prevalence between 2022 and 2042 (Figure 1a). HIV incidence was also projected to decline in all models and by a greater magnitude in Thembisa (Figure 1b). The modelled HIV care cascade in 2022 was similar in Synthesis and Thembisa with mean 76.1% [95% CI 74.1, 78.0] and 71.5% [71.5, 71.6] of people living with HIV (PLWH) on ART, respectively, and mean 70.2% [67.6, 72.8] and 66.2% [66.1, 66.3] of PLWH virally suppressed, respectively. EMOD‐HIV assumed higher ART coverage (81.4% [81.2, 81.5]) but viral suppression (66.5% [66.3, 66.7]) similar to Thembisa (Figure 1c). EMOD‐HIV assumed the highest PrEP coverage in 2022 (2.2% [2.19, 2.20]) compared to 0.8% [0.6, 0.9] (Synthesis) and only 0.05% [0.0496, 0.0498] (Thembisa) (Figure S3.2).

### 3.2 HIV incidence concentration assumed in each model

EMOD‐HIV assumed that people in the high‐ and medium‐exposure group had nine and six times higher likelihood of HIV acquisition compared to the low‐exposure group, respectively. Thembisa's high‐exposure group had five times higher likelihood of HIV acquisition compared to the low‐exposure group. Synthesis created exposure groups differently at each PrEP coverage level and estimated that people in the high‐exposure group had 34 times higher likelihood of HIV acquisition than in the low‐exposure group with 5% PrEP coverage (see Supporting Information for full details).

EMOD‐HIV and Thembisa estimated that 60–65% of the HIV incidence was concentrated into a quarter of the population, with the remaining incidence distributed in the low‐exposure group (Figure 1d). In contrast, Synthesis assumed highly concentrated HIV risk distribution with 73% of the HIV incidence allocated into 8% of the population in the 5% PrEP coverage scenario.

### 3.3 PrEP expansion scenarios

**Figure 2:**
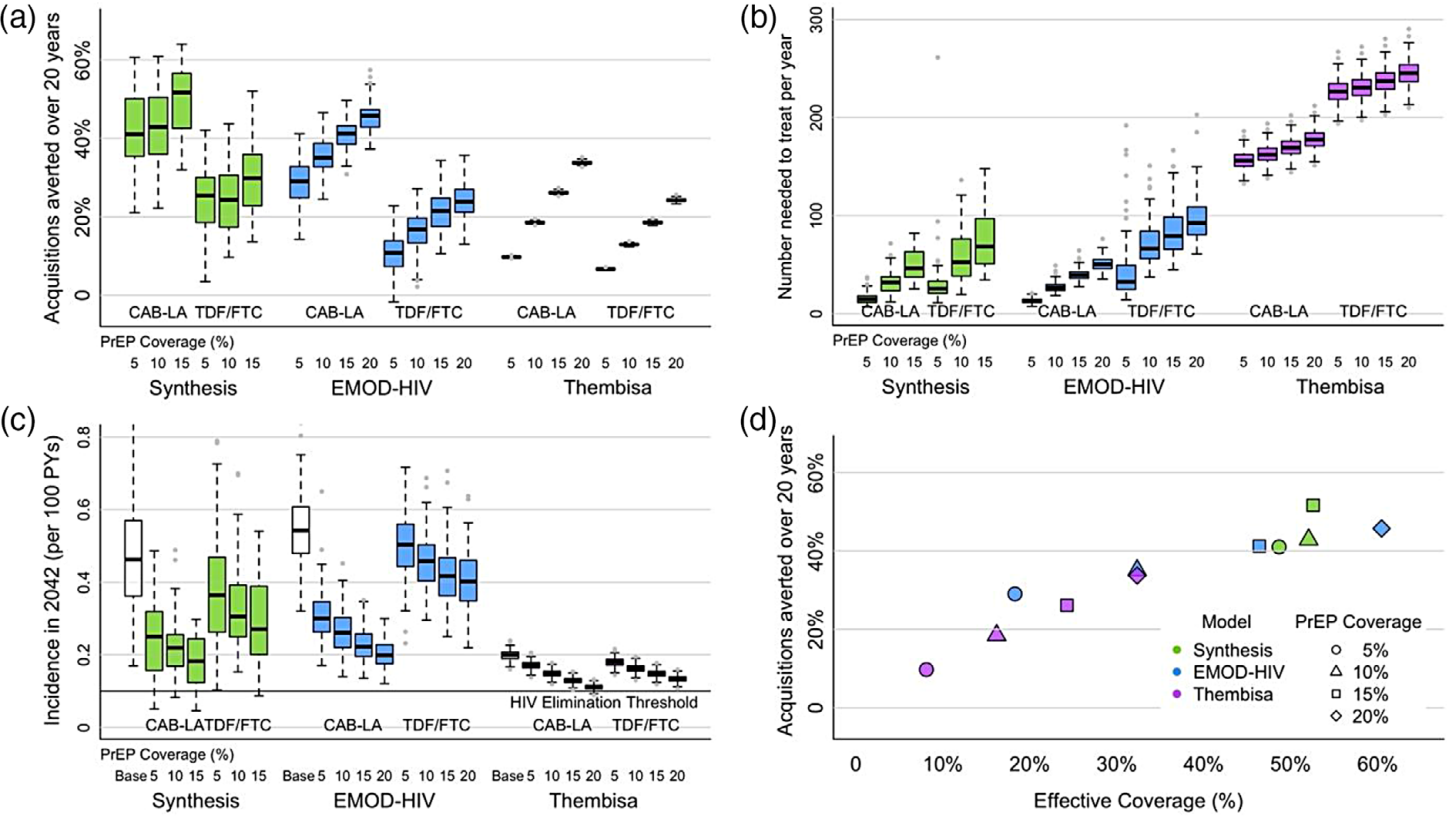
**Projected impact of prioritized PrEP expansions among women and men. Population impact, efficiency, progress to the elimination threshold and proportion of effective coverage achieved**. (a) Population impact in terms of proportion of HIV acquisitions averted from 2022 to 2042 with different PrEP coverage targets achieved by 2032. (b) Population efficiency in terms of additional years on PrEP needed to prevent one acquisition from 2022 to 2042. (c) HIV incidence in 2042 showing progress towards HIV elimination threshold of 0.1/100 PYs. White bars show incidence with baseline PrEP coverage. (d) Median proportion of HIV acquisitions averted with CAB‐LA from 2022 to 2042 with estimated proportion of effective coverage with different PrEP expansions, when PrEP coverage targets are met in 2032. (a−c): Dotted lines show maximum/minimum without outliers. Abbreviations: CAB‐LA, long‐acting injectable cabotegravir; HIV, human immunodeficiency virus; PrEP, pre‐exposure prophylaxis; PYs, person‐years; TDF/FTC, tenofovir disoproxil fumarate/emtricitabine.

Expanding CAB‐LA coverage to 5% of all South African adults without HIV, prioritized to those in high‐exposure groups by 2032, would avert a mean of 42.6% [95% CI 40.1, 45.2] (Synthesis), 29.0% [27.9, 30.0] (EMOD‐HIV) and 9.8% [9.7, 9.8] (Thembisa) of HIV acquisitions over 20 years (Figure 2a). Increasing CAB‐LA coverage to 15% increased acquisitions averted to 49.8% [45.9, 53.8] (Synthesis), 40.9% [40.1, 41.6] (EMOD‐HIV) and 26.2% [26.1, 26.3] (Thembisa). Expanding PrEP coverage to 5% with TDF/FTC instead of CAB‐LA had less impact in all models, with 23.4% [21.0, 25.9] (Synthesis), 10.6% [9.5, 11.6] (EMOD‐HIV) and 6.7% [6.65, 6.70] (Thembisa) acquisitions averted. Meeting coverage targets 5 years earlier (by 2027) with 5% CAB‐LA PrEP coverage increased acquisitions averted to 46.3% [42.9, 49.7] (Synthesis), 34.7% [33.9, 35.6] (EMOD‐HIV) and 12.3% [12.2, 12.3] (Thembisa, Figure S3.3).

The number of individuals NNT to prevent one HIV acquisition with 5% CAB‐LA expansion was substantially lower in Synthesis (16.2 [14.3, 18.1]) and EMOD‐HIV (13.1 [12.5, 13.6]) than in Thembisa (156.9 [155.2, 158.7], Figure 2b) due to more successful exposure‐targeted PrEP use assumed, combined with significantly lower overall HIV incidence projected in Thembisa. If TDF/FTC was used instead of CAB‐LA, NNT would increase to 28.4 [13.8, 42.9] (Synthesis), 39.8 [5.4, 74.1] (EMOD‐HIV) and 227.3 [224.8, 229.7] (Thembisa) individuals to prevent one acquisition. Increasing the PrEP coverage target was less efficient across models due to a reduction in the potential remaining acquisitions averted combined with more PrEP offered to low‐exposure groups.

We measured progress towards HIV elimination by comparing estimated HIV incidence over time with the WHO suggested threshold of 0.1/100 PYs. For 5% CAB‐LA expansion, all models projected significant HIV incidence reduction, down to 0.25/100 PYs [0.22, 0.28] (Synthesis), 0.30/100 PYs [0.29, 0.32] (EMOD‐HIV) and 0.17/100 PYs [0.169, 0.173] (Thembisa) in 2042 (Figure 2c). Increasing PrEP coverage to 20% resulted in HIV incidence that approached the threshold in Thembisa (0.11/100 PYs [0.109, 0.112] with CAB‐LA, 0.13/100 PYs [0.131, 0.135] with TDF/FTC), which was also the most optimistic in the baseline projections. The expected incidence in the other two models remained above the threshold at 0.20/100 PYs [0.19, 0.21] (EMOD‐HIV with 20% CAB‐LA coverage) and 0.18/100 PYs [0.15, 0.21] (Synthesis, with 15% CAB‐LA coverage) suggesting HIV prevention gaps that need to be filled by other interventions.

Each model had different effective coverage resulting from the same levels of overall population PrEP coverage (Figure 2d). With a low baseline PrEP coverage of 0.8% (Synthesis) and 2.2% (EMOD‐HIV), the estimated effective coverage was 7.4% (Synthesis) and 9.9% (EMOD‐HIV) before the start of PrEP expansion in 2022. Thembisa assumed almost no PrEP use in 2022 resulting in negligible effective coverage. In Synthesis, 5% and 15% absolute PrEP coverage was associated with 48.6% and 52.6% effective coverage, respectively, as the group indicated for PrEP use grew when higher overall coverage was targeted, allowing people with a lower likelihood of HIV acquisition to start PrEP. This explained the high impact of the 5% PrEP expansion and the limited additional benefits of achieving 10% and 15% coverage targets. In comparison, EMOD‐HIV achieved only 18.3% effective coverage with a 5% expansion but steadily increased with higher coverage targets. Fifteen percent PrEP expansion with EMOD‐HIV achieved comparable effective coverage as 5% PrEP expansion with Synthesis and showed comparable impact. With less precise prioritization assumed, PrEP expansions simulated by Thembisa achieved lower effective coverage than the other two models, resulting in less impactful interventions. Again, scenarios with similar effective coverage across models (20% expansion in Thembisa vs. 10% expansion in EMOD‐HIV) projected similar impact in terms of HIV acquisitions averted. Additional simulations with EMOD‐HIV in the absence of PrEP showed dynamic trends in the proportions of HIV incidence associated with different exposure groups (Figure S3.1).

### 3.4 Offering PrEP to women only may limit the potential impact of the intervention

**Figure 3:**
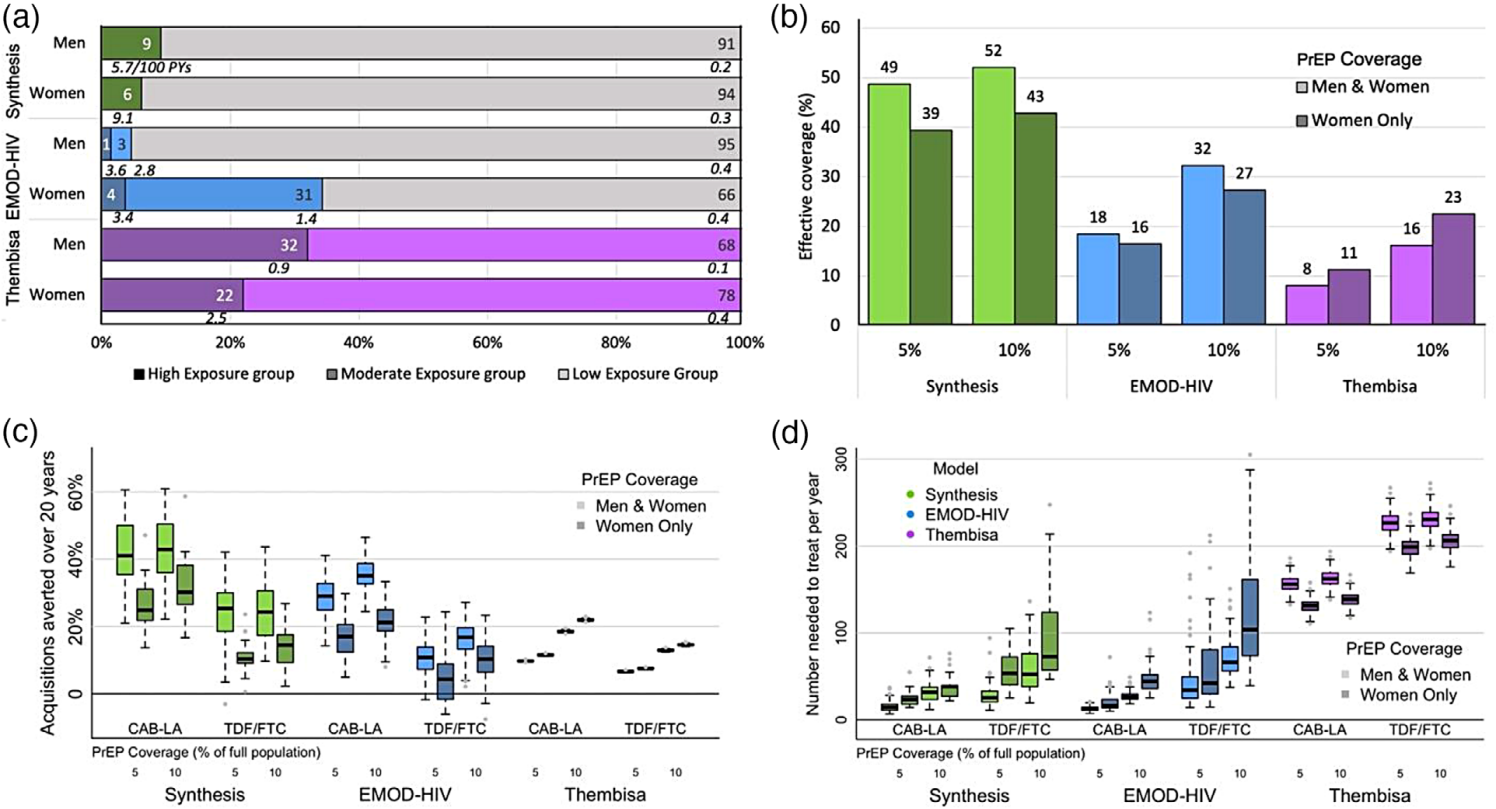
**Comparison of PrEP expansion among women and men versus women only**. (a) Group sizes by gender in 2032 with incidence level (per 100 PYs) in each group shown in italics. 5% PrEP coverage scenario shown for Synthesis; with 10% PrEP coverage, 12% of women and 17% of men are in the high‐exposure group. (b) Effective coverage for different PrEP expansions, estimated as the PrEP coverage of each exposure group weighted by the proportion of new acquisitions projected to occur in that group. (c) Acquisitions averted when PrEP expansion is prioritized to women and men (lighter colours) or to women only (darker colours). (d) Population efficiency as additional years on PrEP needed to prevent one acquisition when expanding to women and men or to women only. Dotted lines show maximum/minimum without outliers. Note that in “women only” scenarios, 5% and 10% coverage of the full population is achieved by covering 10% and 20% of all women, respectively. Abbreviations: CAB‐LA, long‐acting injectable cabotegravir; PrEP, pre‐exposure prophylaxis; TDF/FTC, tenofovir disoproxil fumarate/emtricitabine.

In the simulated scenarios above, PrEP was offered to both women and men. Next, we evaluated PrEP expansion among women only in which the total amount of PrEP used is similar in both scenarios (representing approximately the same total PrEP doses provided). We compared scenarios achieving a specific coverage level (e.g. 5%) in both women and men to corresponding scenarios with twice the coverage (e.g. 10%) in women, for the same level of total population coverage. Notably, women and men made up different proportions of the HIV exposure groups in each model (Figure 3a). In Synthesis, 6% of women and 9% of men were in the high‐exposure group when 5% PrEP coverage is targeted and 12% of women and 17% of men were in the high‐exposure group when 10% PrEP coverage is targeted. In Thembisa, 22% of women and 32% of men were included in the high‐exposure group. In both models, HIV incidence of the high‐exposure group women was higher than that of high‐exposure group men (9.1/100 PYs vs. 5.7/100 PYs in Synthesis with 5% PrEP coverage, 5.3/100 PYs vs. 3.0/100 PYs in Synthesis with 10% PrEP coverage and 2.5/100 PY vs. 0.9/100PYs in Thembisa). In contrast, in EMOD‐HIV, 34% of women and only 5% of men were in the high‐ or medium‐exposure groups and the assumed HIV incidence was similar between genders (3.4/100 PYs for women and 3.6/100 PYs for men).

The Synthesis and EMOD‐HIV models predicted higher effective coverage if PrEP was offered to all than to women only: 5% PrEP expansions in both women and men were estimated to result in 10 percentage points (pp) and 2pp higher effective coverage, respectively. In contrast, Thembisa predicted that effective coverage would increase by 3pp if PrEP was offered to women only (Figure 3b). In the EMOD‐HIV and Thembisa models, the difference between PrEP expansions to all and to women grew larger (to 5pp and 7pp, respectively) when PrEP coverage increased to 10%, while the difference remained similar in Synthesis.

Scenarios with a higher proportion of effective coverage resulted in more acquisitions averted. CAB‐LA expansion covering 10% of women only was projected to have substantially lower acquisitions averted in Synthesis (26.4% [23.2, 29.5]) and EMOD‐HIV (16.5% [15.5, 17.5]) compared to CAB‐LA expansion covering 5% of men and women (Figure 3c). In contrast, Thembisa projected a comparable impact in these scenarios marginally favouring PrEP expansion in women only (11.6% [11.5, 11.6]) where the effective coverage was slightly higher. Results were qualitatively similar when PrEP expansion was performed with TDF/FTC, with lower projected impact across all scenarios. The efficiency of PrEP expansions with NNT when 5% CAB‐LA expansion prioritized women only was also higher than scenarios offering PrEP to all in Synthesis (24.6 [20.9, 28.4]) and EMOD‐HIV (20.0 [18.1, 21.8]) but lower in Thembisa (131.5 [130.0, 133.1], Figure 3d).

### 3.5 Expanding PrEP coverage without prioritization will likely reduce intervention impact

EMOD‐HIV and Thembisa simulated expanding PrEP coverage proportionally throughout the entire population instead of prioritizing it to the populations experiencing high HIV incidence. Achieving 5% proportional coverage was projected to have substantially lower acquisitions averted (CAB‐LA: 0.8% [−0.4, 2.0], TDF/FTC: −2.4% [−3.8, −1.0]) by EMOD‐HIV, which assumes more concentrated HIV incidence. In comparison, acquisitions averted with 5% proportional PrEP distribution (CAB‐LA: 6.7% [6.6, 6.7], TDF/FTC: 4.58% [4.56, 4.59]) were more similar to the prioritized distribution in Thembisa, which assumes more homogeneous HIV incidence distribution (Figure S3.4). These results reflect differences in effective coverage, which were larger for EMOD‐HIV (15pp and 39pp for scenarios with 5% and 20% PrEP coverage, respectively) and smaller for Thembisa (3pp and 12pp for scenarios with 5% and 20% PrEP coverage, respectively, Figure S3.5).

## 4 DISCUSSION

Highly effective long‐acting PrEP with CAB‐LA could dramatically reduce HIV incidence in South Africa. Using three epidemic models, we projected the impact of expanding PrEP use on the HIV epidemic in South Africa with CAB‐LA or TDF/FTC. Each model predicted that expanding PrEP access with CAB‐LA will have a much larger impact on preventing HIV acquisitions than an expansion with the same coverage of TDF/FTC, as the lack of regular pill taking needed with CAB‐LA results in higher effectiveness [1, 2]. Projected impact differences could be multiplied further if CAB‐LA expansions result in higher population coverage, which seems likely based on preference data [3, 4, 5], and if expansions included both CAB‐LA and TDF/FTC, allowing individuals their choice of regimens. By including three independently developed and calibrated models and endeavouring to compare results and explain discrepancies, we presented three different PrEP distribution policies, epidemic settings and model structures side by side as different pieces of the same puzzle, which in combination may better inform future decisions on PrEP expansion. We leave readers to decide which modelling approach most closely relates to their own epidemic conditions and which intervention scenario is most feasible for their implementation. In addition, we proposed a new metric (effective coverage) which is a good predictor of intervention success across models and could serve as a unifying indicator when interventions are evaluated and compared.

As the pace of PrEP expansion in South Africa is uncertain, we provided projections on PrEP impact across a range of assumptions about uptake. In general, greater population coverage resulted in more acquisitions averted. However, we found that effective coverage, an alternative metric that weights the PrEP coverage of sub‐populations by their respective rates of exposure, was more predictive of epidemic impact. One key observation is that scenarios with similar effective coverage resulted in comparable impact estimates across models, highlighting the importance of providing HIV prevention to populations that experience high HIV incidence. Each model suggested unique prevention approaches based on different assumptions regarding the concentration of HIV incidence within sub‐populations defined by age, gender and sex work. They also employed different mechanisms of how well PrEP use can be prioritized to those who need it most, which encompasses both correct (self‐) assessment of HIV acquisition risk and additional motivation for PrEP uptake. This is a strength of the model comparison analysis, in which different models inform different pieces of the HIV prevention puzzle as the actual reach and uptake of future CAB‐LA PrEP programmes is unknown. We demonstrated that interventions that specifically prioritize key populations with disproportionate attributable fractions of HIV incidence would be very efficient, but we realize implementation may be challenging. The proposed effective coverage metric, which requires a proper assessment of HIV incidence distribution, could be informative in planning well‐prioritized PrEP interventions.

Another important consideration when utilizing CAB‐LA in South Africa is whether to make it available to all people or only to women. Synthesis and EMOD‐HIV assumed highly concentrated HIV incidence within a small proportion of women and men and showed substantial gains when both women and men were offered PrEP, emphasizing the importance of including men in PrEP programmes in South Africa. In these models, PrEP expansion among women beyond the high‐exposure group is less advantageous than offering PrEP to highly exposed men. This pattern was not confirmed by Thembisa, which assumed significantly higher HIV incidence in women compared to men and less granular assessment of HIV risk, placing more than 22% of women in the high HIV exposure group. This resulted in a comparable population impact of both prioritizations with a slight preference to women‐only PrEP scenarios.

Our analysis suggests that adding CAB‐LA is unlikely to be sufficient to end the HIV epidemic in South Africa. Even interventions assuming consistent CAB‐LA use by a large proportion (20%) of the population without HIV, successfully prioritized to high HIV incidence populations, were not enough to reduce HIV incidence below the WHO suggested threshold of 0.1/100 PYs. This was true even for Thembisa which projected a low HIV incidence of 0.2/100 PYs in the baseline scenario without PrEP expansion. In that sense, our analysis identified HIV prevention gaps which need to be filled, for example, by focusing attention to social determinants of health and structural barriers that limit access to PrEP, to other existing biomedical prevention options like the dapivirine ring, condoms and medical male circumcision, and to continuing to pursue novel prevention tools, such as effective and affordable HIV vaccines.

As with any modelling study, this analysis has several limitations. Each model determined PrEP allocations within their populations independently, which did not allow for creating ensemble estimates. The analysis also projected the impact of PrEP expansions to pre‐specific PrEP coverage levels but does not discuss the feasibility of reaching and sustaining those targeted levels for the proposed prioritization strategies. Presented results are largely based on theoretical risk assessment rather than integration of structural barriers or social determinants of health in models. Only Thembisa included MSM and no models included transgender populations despite their disproportionately high HIV incidence [31] due to data limitations as, until recently, transgender population data was generally aggregated with MSM data.

## 5 CONCLUSIONS

CAB‐LA is an important new PrEP modality that could significantly reduce the number of HIV acquisitions in South Africa based on projections from three models. Care will need to be taken to incorporate information about how HIV incidence is distributed across the population to maximize the impact of PrEP expansion while ensuring equity of access to effective and acceptable prevention options. We demonstrated that effective coverage, as defined in this analysis, may serve as a vital metric in evaluating the success of PrEP or other HIV prevention interventions.

## COMPETING INTERESTS

The authors have no conflicts of interest to declare.

## AUTHORS’ CONTRIBUTIONS

Conception of the research question: SES, DJD, M‐CB and AP. Model development, parameterization, coding and simulation for Synthesis: AP, JS, VC and LB‐M; for EMOD‐HIV: AB and DK; Thembisa: LJ, GM‐R and LFJ. Model comparison analysis, statistical analysis, figure and table creation: SES. Drafting of the manuscript: SES, MM and DJD. Critical input into draft manuscript: all authors. Read and approved the final manuscript: all authors.

## FUNDING

Funding for SES, MM, JH, RVB, M‐CB and DJD was provided by the NIH NIAID (grant number UM1 068617). Funding for SES, DJD and MM was provided by the NIH NIAID (grant number R01 AI179417). Funding for LJ, GM‐R and LFJ was provided by the Bill and Melinda Gates Foundation (grant number 019496). Funding for the HIV Modelling Consortium (AP, JS, VC, LB‐M, DK and AB) was provided by the Bill and Melinda Gates Foundation (grant number INV‐007145). Funding for VC was provided by the Medical Research Council (grant number MR/T042796/1). Funding for M‐CB was provided by the MRC Centre for Global Infectious Disease Analysis (reference MR/X020258/1), funded by the UK Medical Research Council (MRC). This UK‐funded award is carried out in the frame of the Global Health EDCTP3 Joint Undertaking.

## Supporting information




**Figure S1.1** Model comparison schematic.
**Figure S2.1**: Graphical diagram of Synthesis model.
**Figure S1.2** Effective coverage schematic.
**Figure S2.2**: Graphical diagram of EMOD‐HIV model (from https://www.idmod.org/tool/emod‐hiv/).
**Figure S2.3**: Graphical diagram of EMOD‐HIV model.
**Figure S2.4**: Graphical diagram of Thembisa model.
**Figure S3.1**: Proportions of acquisitions from each exposure group in EMOD‐HIV when no PrEP is included in the model.
**Figure S3.2**: Baseline model projections by Synthesis (green), EMOD‐HIV (blue) and Thembisa (purple).
**Figure S3.3**: Projected impact of prioritized PrEP expansions on population effectiveness among men and women.
**Figure S3.4**: PrEP expansion among men and women versus women only and prioritized to those PrEP‐eligible (left) versus proportionally among all people not living with HIV (right).
**Figure S3.5**: PrEP expansion prioritized to those PrEP‐eligible versus proportionally among all people not living with HIV.
**Table S2.1**: Model characteristics.
**Table S2.2**: Synthesis PrEP eligibility criteria.

## Data Availability

Data will be shared upon request.
