# Supplementary material for: Estimated impact of long‐acting injectable PrEP in South Africa: a model comparison analysis
Source: J Int AIDS Soc. 2025 Jul 2;28(Suppl 2):e26453. doi: 10.1002/jia2.26453 (PMC12215805; doi:10.1002/jia2.26453)
Supplement: Supplementary file 1 — Figure S1.1 Model comparison schematic. Figure S2.1: Graphical diagram of Synthesis model. Figure S1.2 Effective coverage schematic. Figure S2.2: Graphical diagram of EMOD‐HIV model (from https://www.idmod.org/tool/emod‐hiv/). Figure S2.3: Graphical diagram of EMOD‐HIV model. Figure S2.4: Graphical diagram of Thembisa model. Figure S3.1: Proportions of acquisitions from each exposure group in EMOD‐HIV when no PrEP is included in the model. Figure S3.2: Baseline model projections by Synthesis (green), EMOD‐HIV (blue) and Thembisa (purple). Figure S3.3: Projected impact of prioritized PrEP expansions on population effectiveness among men and women. Figure S3.4: PrEP expansion among men and women versus women only and prioritized to those PrEP‐eligible (left) versus proportionally among all people not living with HIV (right). Figure S3.5: PrEP expansion prioritized to those PrEP‐eligible versus proportionally among all people not living with HIV. Table S2.1: Model characteristics. Table S2.2: Synthesis PrEP eligibility criteria. [file JIA2-28-e26453-s001.docx]

**Supplementary Information: Estimated impact of long-acting injectable PrEP in South Africa: A model comparison analysis**

Sarah E. Stansfield, Mia Moore, Lise Jamieson, Gesine Meyer-Rath, Leigh F. Johnson, David Kaftan, Anna Bershteyn, Jennifer Smith, Valentina Cambiano, Loveleen Bansi-Matharu, Andrew Phillips, Jesse Heitner, Ruanne V. Barnabas, Brett Hanscom, Deborah J. Donnell, Marie-Claude Boily, Dobromir Dimitrov

**1. Expanded Methods**

**1.1 Definition of effective PrEP coverage (Equation S1)**

PrEP_high = PrEP coverage in the high exposure group

PrEP_mod = PrEP coverage in the moderate exposure group

PrEP_low = PrEP coverage in the low exposure group

Acq_high = % of new HIV acquisitions that occur in the high exposure group

Acq_high = (Incidence in the high exposure group) * (Proportion of population in high exposure group)/(Incidence in the full population)

Acq_mod = % of new HIV acquisitions that occur in the moderate exposure group

Acq_mod = (Incidence in the moderate exposure group) * (Proportion of population in moderate exposure group)/(Incidence in the full population)

Acq_low = % of new HIV acquisitions that occur in the low exposure group

Acq_low = (Incidence in the low exposure group) * (Proportion of population in low exposure group)/(Incidence in the full population)

**Effective coverage** = (PrEP_high * Acq_high + PrEP_mod * Acq_mod + PrEP_low * Acq_low)*100

**1.2 Model comparison collaboration**

The HPTN Modelling Centre (<https://hptnmodelling.org/>) and the HIV Modelling Consortium (http://hivmodeling.org/) initiated a collaboration to project population-level impacts of PrEP scale up, including CAB-LA, by inviting teams to participate in model comparison projects, using specific model parameters and scenarios. In this project, we showed that offering CAB-LA to MSM populations can help end the HIV epidemic in high-income countries only if it increases overall PrEP coverage. Such PrEP expansions were projected to be more efficient in settings with high HIV incidence.^24^

**Figure 1.1** Model comparison schematic

**1.3 PrEP usage**

Each model provided differential PrEP access to different exposure groups. In EMOD-HIV, PrEP was initially expanded among those in the high exposure group (as defined in the Supplement), only expanding to the medium exposure group when needed to increase coverage levels. In Thembisa, PrEP was available to all individuals, but people from the high exposure group were three times more likely to initiate PrEP compared to the rest of the population. Finally in Synthesis, PrEP expansion was prioritized according to one of ten different sets of criteria involving gender, partner HIV status, and sexual activity. Simulations with all ten prioritization sets were sorted by the overall population PrEP coverage achieved in each stochastic run (see below).

**1.4 PrEP scenarios**

Each model simulated a baseline scenario over 20 years assuming: 1) TDF/FTC use remained at baseline level, and 2) ART coverage remained near baseline level (76% Synthesis, 81% EMOD-HIV) or expanded over time (from 72% to 78% Thembisa).

PrEP expansion scenarios varied the magnitude and pace of expansion as well as whether PrEP was prioritized to high exposure groups. PrEP expansion was initiated in 2022 and increased the overall PrEP coverage from baseline to between 5-20% (5-15% Synthesis) of the total adult population in South Africa without HIV (Table 2). The pace of PrEP expansion was varied to hit coverage targets within 5 or 10 years. Simulations were constrained to not exceed the targeted coverage by more than 1 percentage point (pp). PrEP expansion occurred by offering either only CAB-LA or TDF/FTC daily pills to all users. In the Synthesis and EMOD-HIV models, PrEP expansion with CAB-LA began with some users on TDF/FTC; <2% of PrEP users remained on TDF/FTC by 2027 (Synthesis) or all users transitioned to CAB-LA when offered (EMOD-HIV). Two interventions were simulated: i) prioritized expansion (main analysis) in which PrEP is offered preferentially to high HIV exposure groups as defined in each model, and ii) proportional expansion, assuming equal likelihood to initiate PrEP across the entire population without HIV. Because HIV prevalence is higher in women compared to men in South Africa,^28^ we performed separate analyses of offering PrEP to cisgender women only versus offering it to both cisgender women and cisgender men (hereafter referred to as women and men), applying the same population coverage targets.

**1.5 HIV incidence** **concentration assumed in each model**

EMOD-HIV estimated that 2.6% of people without HIV were in the high exposure group and 17.4% were in the medium exposure group, with 9- and 6-times higher likelihood of HIV acquisition compared to the low exposure group, respectively. Thembisa assumed more uniform risk distribution with 27.0% of people without HIV forming the high exposure group, with 5 times higher likelihood to acquire HIV compared to the remaining population in the low exposure group. Synthesis created exposure groups differently at each PrEP coverage level (see Supplement for full details) and estimated that, in the scenarios with 5% PrEP coverage, 7.5% of people without HIV on average were in the high exposure group at any time and the likelihood of HIV acquisition was approximately 34 times higher than in the low exposure group. In the 10% and 15% PrEP coverage scenarios, 14.5% and 24.2% of people were in the high exposure group, respectively, with estimated 18- and 17- times higher likelihood of HIV acquisition compared to the low exposure group, respectively.

EMOD-HIV and Thembisa estimated that 60-65% of the HIV incidence was concentrated into a quarter of the population, with the remaining incidence almost identically distributed in the low exposure group for each model (Fig 1D). In comparison, Synthesis assumed highly concentrated HIV risk distribution with 73% of the HIV incidence allocated into 8% of the population in the 5% PrEP coverage scenario.

**1.6 Effective coverage**

**Figure S1.2** Effective coverage schematic

**2. Additional model descriptions**

**Table S2.1:** Model characteristics

| **Model Characteristics** | **Synthesis** | **EMOD-HIV** | **Thembisa** |
| --- | --- | --- | --- |
| Model structure | Stochastic individual-based | Stochastic individual-based | Deterministic compartmental |
| Modeled population | Heterosexuals, FSW | Heterosexuals, FSW | Heterosexuals, FSW, men who have sex with men (MSM) |
| Population attributes/stratification | Individual gender, age, sexual behavior, male circumcision status | Individual gender, age, sexual behavior, male circumcision status | Gender, age, sexual behavior, male circumcision status |
| Age range | 15+ | 0-90^1^ | 0-90^1^ |
| Baseline TDF/FTC coverage | 0.76% | 2.20% | 0.05% |
| TDF/FTC effectiveness | Mean 70%^2^ | 58% | 85% MSM  65% remaining population |
| TDF/FTC discontinuation rate^3^ | 0.01-0.05 per 3 months | 0.31 per 3 months | 0.021 per 3 months |
| CAB-LA effectiveness | 95% (80% runs)  90% (20% runs) | 95% | 91% men  95% women |
| CAB-LA discontinuation rate^3^ | 0.01-0.05 per 3 months | 0.021 per 3 months | |
| Proportion in each HIV exposure group^4^ | High exposure: 8%, 16%, 24% of adults age 15-65^5^ with 5%, 10%, and 15% PrEP coverage respectively | High exposure: 3%  Medium exposure: 17%  Low exposure: 80% | High exposure group:  women: 22%  men: 32% |
| HIV risk ratio between exposure groups^6,7^ | 34x, 18x, 17x higher in high exposure vs low exposure group^5^ with 5%, 10%, and 15% PrEP coverage respectively | Low: Medium: High exposure group  1:6:9 | 5x higher in high exposure vs low exposure group |
| PrEP allocation by HIV exposure group  (Prioritization model scenarios) | PrEP only used by those in the high exposure group in any given 3-month period^5^ | High exposure group covered before any of the medium exposure group is covered | High exposure group 3x more likely to take up PrEP than low exposure group |
| Number of replicates | 23 to 53 (median 43) | 100 | 100 |
| Viral suppression definition | Viral load (VL)<1000 copies/ml | Not modeled directly, when transmission risk approaches 0 labeled virally suppressed | VL<400 copies/ml |
| Condom use with PrEP | PrEP only used with condomless sex | No interaction between PrEP and condom use | PrEP users slightly decrease condom use |
| Calibration | HIV prevalence data, ART coverage, male circumcision rates, mortality data | HIV prevalence data, population data, and ART coverage | HIV prevalence data, mortality data, ART coverage data and age distributions of ART patients |
| Validation | Incidence data held out from model fitting data | Incidence data held out from model fitting data | Estimates of HIV incidence from national household surveys, antenatal surveys and a national survey of sex workers (these incidence estimates were not used in calibration) |

^1^ All calculations (PrEP coverage, etc.) were based on the population 15 years and older.

^2^ Effectiveness was defined as the combination of product efficacy and adherence.^2^ There was a 20% chance efficacy=90% and an 80% chance efficacy=95%. Adherence was > 80% in 90% of individuals, resulting in mean effectiveness of approximately 70%.

^3^ Discontinuation rates despite continued risk

^4^ HIV exposure groups are defined by differences in condomless sexual activity and age-sex mixing patterns which affect the likelihood to acquire HIV.

^5^ The model uses specific PrEP indication criteria which we use to define the high exposure group. This estimate represents the mean over multiple stochastic runs. (see Appendix).

^6^ Relative difference in annual HIV incidence between groups in the absence of PrEP.

^7^ These are model outputs not inputs.

Abbreviations: FSW: female sex worker; MSM: men who have sex with men; TDF/FTC: tenofovir disoproxil fumarate/emtricitabine; CAB-LA: long acting injectable cabotegravir; HIV: human immunodeficiency virus; PrEP: pre-exposure prophylaxis

***2.1 Synthesis:***

Individuals having an indication for PrEP were determined every three months. Criteria are summarized in Table S2. Eligibility criteria 6, 7, 10, 11, and 13 were for the women-only PrEP scenarios.

5% PrEP coverage of men and women included simulations using criteria 4, 8, 5, and 9.

10% and 15% PrEP coverage of men and women included simulations using criteria 4, 8, 5, 9, and 12.

5% PrEP coverage of women only included simulations using criteria 6, 7, 11, and 13.

10% PrEP coverage of women only included simulations using criteria 13.

*See* Smith J, Bansi-Matharu L, Cambiano V, Dimitrov D, Bershteyn A, van de Vijver D, et al. Predicted effects of the introduction of long-acting injectable cabotegravir pre-exposure prophylaxis in sub-Saharan Africa: a modelling study. The lancet HIV. 2023;10(4):e254-e65 *for further detail on CAB-LA modeling*.

**Table S2.2:** Synthesis PrEP eligibility criteria

| # | Period | Gender | Criteria | Probability of PrEP |
| --- | --- | --- | --- | --- |
| 4 | Current 3-month period | Women or men | Condomless sex with 1+ short-term partner | 100% |
|  | Current 3-month period | Women or men | Condomless sex with long-term known HIV+ partner | 100% |
|  | Current 3-month period | Women | Condomless sex with long-term suspected HIV+ partner (partner truly HIV+) | 50% |
|  | Current 3-month period | Women | Condomless sex with long-term suspected HIV+ partner (partner truly HIV-) | 5% |
| 5 | Current 3-month period **or last 6 months** | Women or men | Condomless sex with 1+ short-term partner | 100% |
|  | Current 3-month period | Women or men | Condomless sex with long-term known HIV+ partner | 100% |
|  | Current 3-month period | Women **or men** | Condomless sex with long-term suspected HIV+ partner (partner truly HIV+) | 50% |
|  | Current 3-month period | Women **or men** | Condomless sex with long-term suspected HIV+ partner (partner truly HIV-) | 5% |
| 8 | Current 3-month period | Women or men | Condomless sex with 1+ short-term partner | 100% |
|  | Current 3-month period | Women or men | Condomless sex with long-term known HIV+ partner | 100% |
|  | Current 3-month period | Women | Condomless sex with long-term suspected HIV+ partner (partner truly HIV+) | 50% |
|  | Current 3-month period | Women | Condomless sex with long-term suspected HIV+ partner (partner truly HIV-) | **10%** |
| 9 | Current 3-month period or last 6 months | Women or men | Condomless sex with 1+ short-term partner | 100% |
|  | Current 3-month period | Women or men | Condomless sex with long-term known HIV+ partner | 100% |
|  | Current 3-month period | Women or men | Condomless sex with long-term suspected HIV+ partner (partner truly HIV+) | 50% |
|  | Current 3-month period | Women or men | Condomless sex with long-term suspected HIV+ partner (partner truly HIV-) | **10%** |
| 12 | Current 3-month period **or last 6 months** | Women or men | Condomless sex with 1+ short-term partner | 100% |
|  | Current 3-month period | Women or men | Condomless sex with long-term partner **(regardless of status)** | 100% |
|  |  |  |  |  |
| Women Only | | | | |
| 6 | Current 3-month period | Women | Condomless sex with 1+ short-term partner | 100% |
|  | Current 3-month period | Women | Condomless sex with long-term known HIV+ partner | 100% |
|  | Current 3-month period | Women | Condomless sex with long-term suspected HIV+ partner (partner truly HIV+) | 50% |
|  | Current 3-month period | Women | Condomless sex with long-term suspected HIV+ partner (partner truly HIV-) | 5% |
| 7 | Current 3-month period or last 6 months | Women | Condomless sex with 1+ short-term partner | 100% |
|  | Current 3-month period | Women | Condomless sex with long-term known HIV+ partner | 100% |
|  | Current 3-month period | Women | Condomless sex with long-term suspected HIV+ partner (partner truly HIV+) | 50% |
|  | Current 3-month period | Women | Condomless sex with long-term suspected HIV+ partner (partner truly HIV-) | 5% |
| 10 | Current 3-month period | Women | Condomless sex with 1+ short-term partner | 100% |
|  | Current 3-month period | Women | Condomless sex with long-term known HIV+ partner | 100% |
|  | Current 3-month period | Women | Condomless sex with long-term suspected HIV+ partner (partner truly HIV+) | 50% |
|  | Current 3-month period | Women | Condomless sex with long-term suspected HIV+ partner (partner truly HIV-) | 10% |
| 11 | Current 3-month period or last 6 months | Women | Condomless sex with 1+ short-term partner | 100% |
|  | Current 3-month period | Women | Condomless sex with long-term known HIV+ partner | 100% |
|  | Current 3-month period | Women | Condomless sex with long-term suspected HIV+ partner (partner truly HIV+) | 50% |
|  | Current 3-month period | Women | Condomless sex with long-term suspected HIV+ partner (partner truly HIV-) | 10% |
| 13 | Current 3-month period **or last 6 months** | Women | Condomless sex with 1+ short-term partner | 100% |
|  | Current 3-month period | Women | Condomless sex with long-term partner (regardless of status) | 100% |

**Figure S2.1: Graphical diagram of Synthesis model**

***2.2 EMOD-HIV:***

There were three exposure groups. The high exposure group included 100% of female sex workers (FSW), 50% individuals in serodiscordant relationships, and 25% of adolescent girls and young women (AGYW). All members of this group were not living with HIV and were in a relationship. The moderate exposure group included male partners of FSW and women under 55 years of age who were not living with HIV and were in a relationship. The low exposure group included the remaining population.


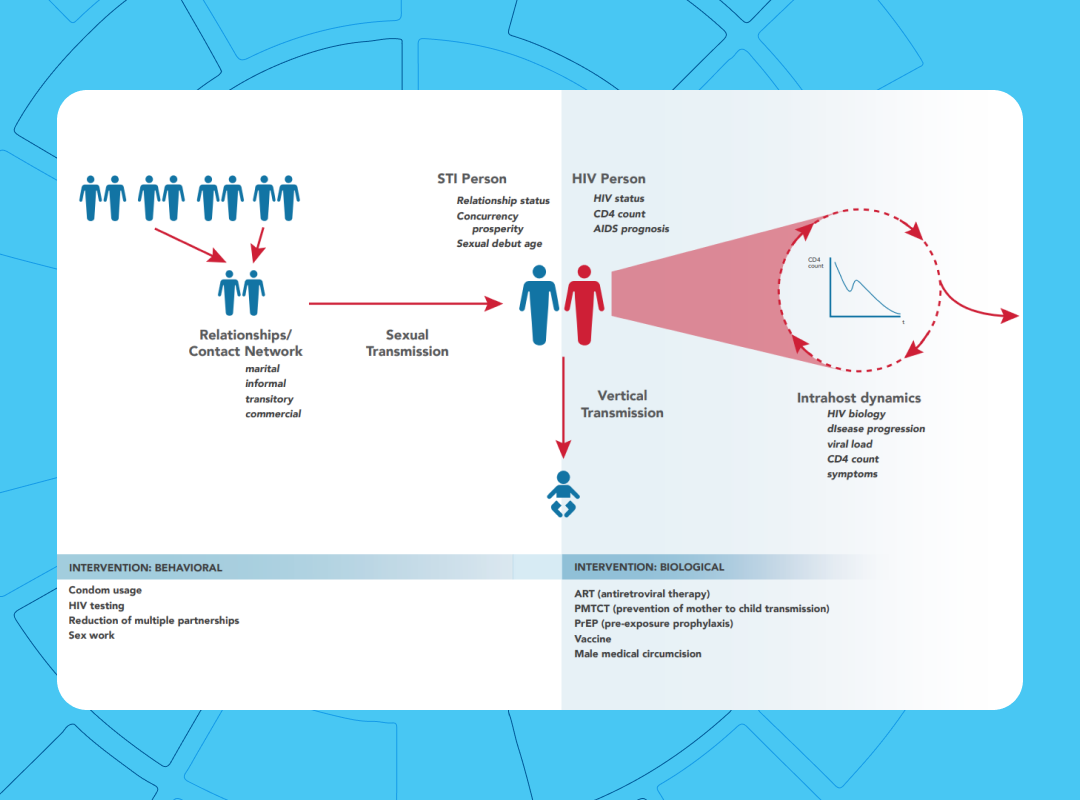


**Figure S2.2: Graphical diagram of EMOD-HIV model *(from*** [***https://www.idmod.org/tool/emod-hiv/***](https://www.idmod.org/tool/emod-hiv/)***)***


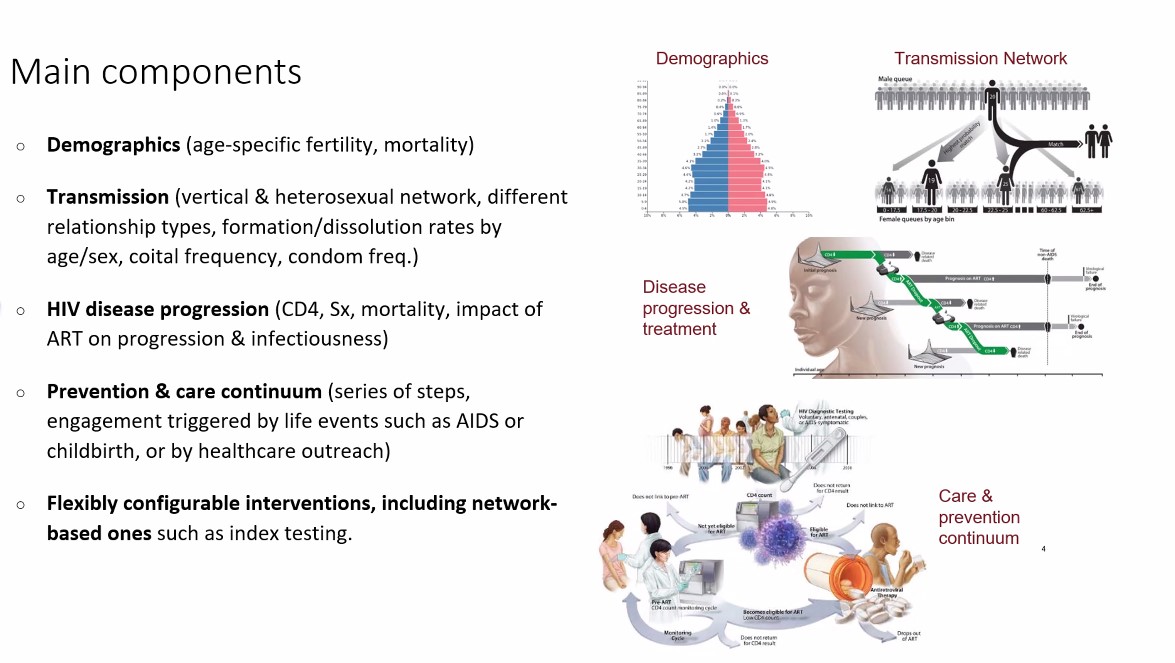


**Figure S2.3: Graphical diagram of EMOD-HIV model**

***2.3 Thembisa:***

The model includes two broad risk groups: high and low (referred to as high and low exposure groups in the main manuscript). The high risk group includes individuals who have a propensity for concurrent partners or commercial sex activity (either as sex workers or as clients). Risk groups are further divided into a number of sub-groups defined in terms of marital status, sexual experience and same-sex activity. Sex workers are modelled as a sub-group within high exposure unmarried women.

Thembisa was calibrated with a Bayesian procedure, setting prior distributions on a number of the key behavioral and biological parameters, and estimating the posterior distribution using Incremental Mixture Importance Sampling. The means and 95% CIs represent posterior means and 95% credibility intervals.

The Thembisa model is a deterministic compartmental model and so does not add stochastic variability when HIV transmission is simulated. Instead it has uncertainty in parameter estimates, sampled from plausible ranges during model calibration. This leads to lower overall variability in Thembisa’s results.

**Figure S2.4: Graphical diagram of Thembisa model**

**3. Additional Figures**


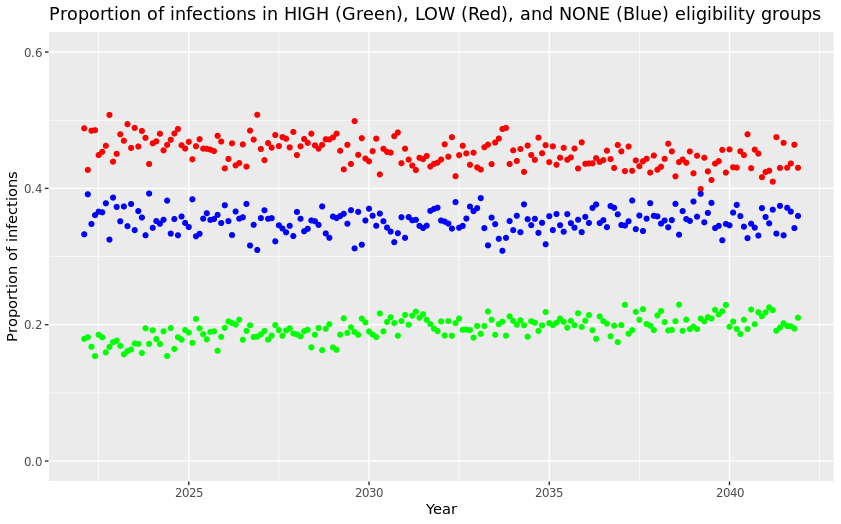


**Figure S3.1: Proportions of acquisitions from each exposure group in EMOD-HIV when no PrEP is included in the model. The high exposure group is in green, the moderate exposure group is in red and the low exposure group is in blue.**


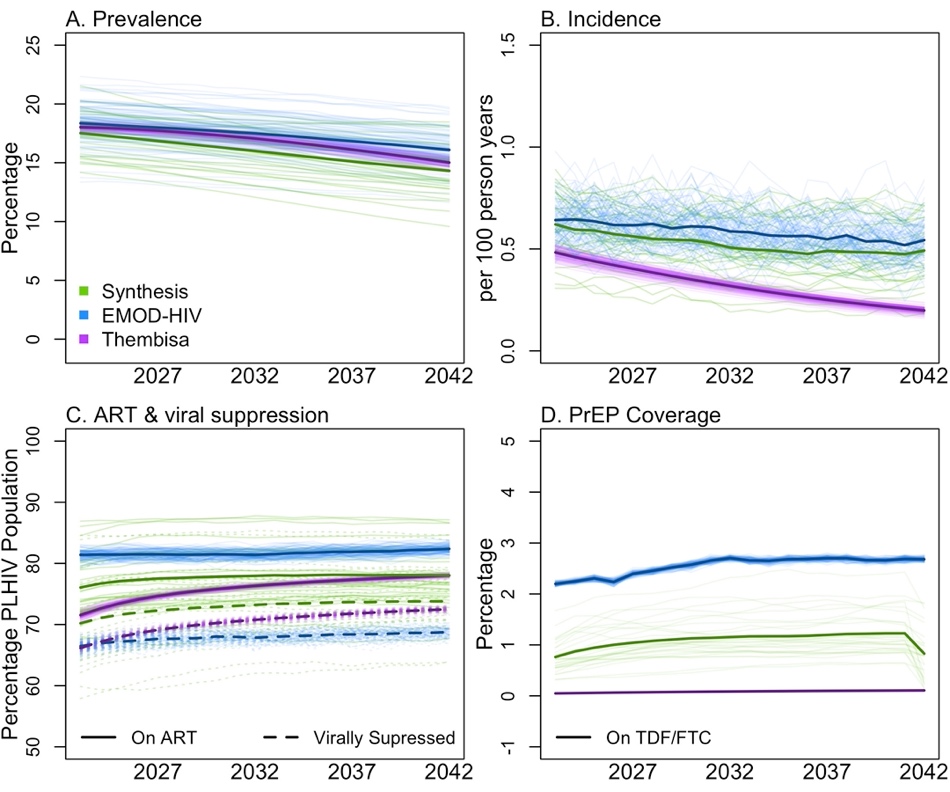


**Figure S3.2: Baseline model projections by Synthesis (green), EMOD-HIV (blue), and Thembisa (purple).** A) HIV prevalence. B) HIV incidence. C) ART and viral suppression among the people living with HIV (PLHIV) population. D) PrEP coverage with TDF/FTC with no PrEP expansion.

**
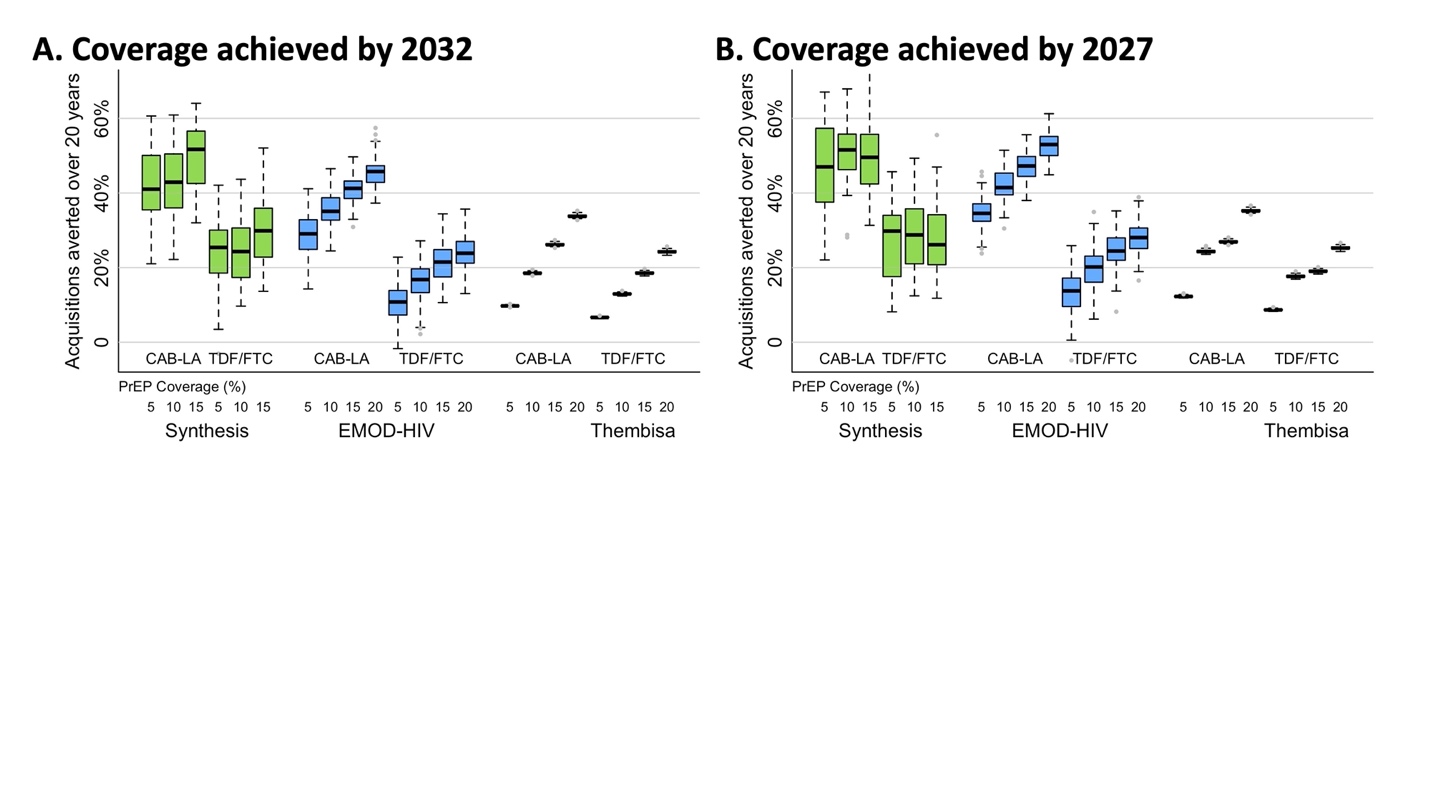
**

**Figure S3.3: Projected impact of prioritized PrEP expansions on** **population effectiveness among men and women.** A: Population effectiveness in terms of proportion of HIV acquisitions averted from 2022-2042 with different PrEP coverage targets achieved by 2032. B: Population effectiveness in terms of proportion of HIV acquisitions averted from 2022-2042 with different PrEP coverage targets achieved by 2027.


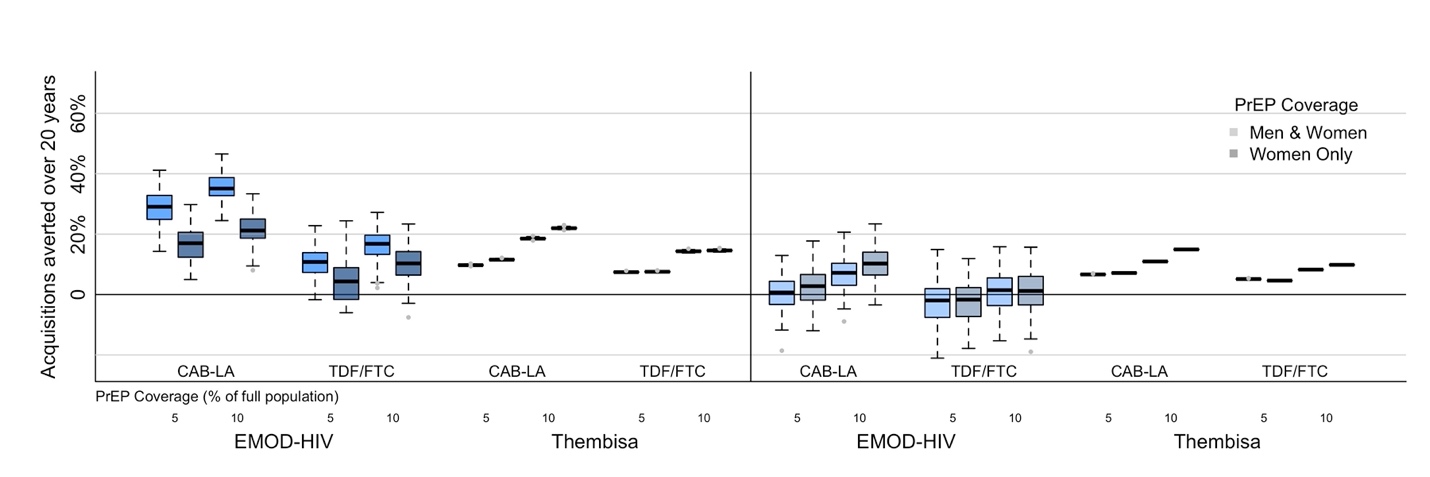


**Figure S3.4: PrEP expansion among men and women versus women only and prioritized to those PrEP-eligible (left) versus proportionally among all people not living with HIV (right).** *5% and 10%* *coverage of the full population in the women only coverage scenario is equal to 10% and 20% coverage of women, respectively.* Acquisitions averted when PrEP expansion is prioritized to those at higher risk (left) or distributed proportionally among the full population not living with HIV (right) and to men and women (lighter colors) or to women only (darker colors). Synthesis did not model proportional coverage of the entire population or 20% coverage of women only. *Notches in boxplot show 95% credible interval for the median.* *Dotted lines show maximum/minimum without outliers.*


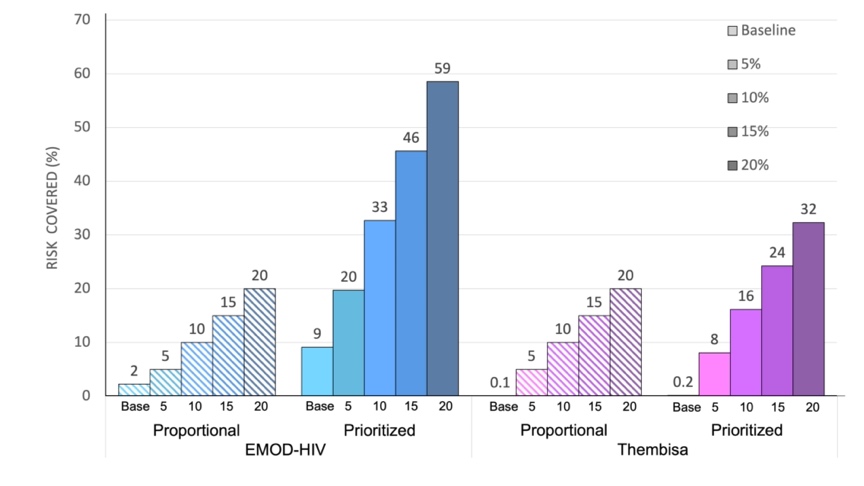


**Figure S3.5: PrEP expansion prioritized to those PrEP-eligible versus proportionally among all people not living with HIV.** Proportion of likelihood of HIV acquisition covered for different PrEP expansions, estimated as the % of 2032 PrEP coverage of each exposure group weighted by the proportion of new acquisitions projected to occur in that group. Synthesis did not model proportional coverage of the entire population.
